# SUMOylation promotes survival and integration of neural stem cell grafts in ischemic stroke

**DOI:** 10.1016/j.ebiom.2019.03.035

**Published:** 2019-03-21

**Authors:** Joshua D. Bernstock, Luca Peruzzotti-Jametti, Tommaso Leonardi, Nunzio Vicario, Daniel Ye, Yang-ja Lee, Dragan Maric, Kory R. Johnson, Yongshan Mou, Aletta Van Den Bosch, Mark Winterbone, Gregory K. Friedman, Robin J.M. Franklin, John M. Hallenbeck, Stefano Pluchino

**Affiliations:** a Stroke Branch, National Institutes of Health (NINDS/NIH), National Institute of Neurological Disorders and Stroke, Bethesda, MD, USA; b Department of Clinical Neurosciences, University of Cambridge, UK; c NIHR Biomedical Research Centre, University of Cambridge, UK; d Department of Biomedical and Biotechnological Sciences, Physiology Section, University of Catania, Italy; e Flow and Imaging Cytometry Core Facility, National Institutes of Health (NINDS/NIH), National Institute of Neurological Disorders and Stroke, Bethesda, MD, USA; f Bioinformatics Section, Information Technology & Bioinformatics Program, Division of Intramural Research (DIR), (NINDS/NIH), Bethesda, MD, USA; g Department of Pediatrics and Neurosurgery, University of Alabama at Birmingham, Birmingham, AL, USA; h Wellcome Trust-Medical Research Council Stem Cell Institute, University of Cambridge, UK

**Keywords:** Neural stem cells (NSCs), SUMOylation, Ubc9, Ischemia/reperfusion, Stroke, Cell therapy, Regenerative medicine, Cellular engineering

## Abstract

**Background:**

Neural stem cell (NSC)-based therapies hold great promise for treating diseases of the central nervous system (CNS). However, several fundamental problems still need to be overcome to fully exploit the clinical potential of NSC therapeutics. Chief among them is the limited survival of NSC grafts within hostile microenvironments.

**Methods:**

Herein, we sought to engineer NSCs in an effort to increase graft survival within ischemic brain lesions *via* upregulation of global SUMOylation, a post-translational modification critically involved in mediating tolerance to ischemia/reperfusion.

**Findings:**

NSCs overexpressing the SUMO E2-conjugase Ubc9 displayed resistance to oxygen-glucose-deprivation/restoration of oxygen/glucose (OGD/ROG) and enhanced neuronal differentiation *in vitro*, as well as increased survival and neuronal differentiation when transplanted in mice with transient middle cerebral artery occlusion *in vivo*.

**Interpretation:**

Our work highlights a critical role for SUMOylation in NSC biology and identifies a biological pathway that can be targeted to increase the effectiveness of exogenous stem cell medicines in ischemic stroke.

**Fund:**

Intramural Research Program of the NINDS/NIH, the Italian Multiple Sclerosis Foundation (FISM), the Bascule Charitable Trust, NIH-IRTA-OxCam and Wellcome Trust Research Training Fellowships.

## Highlights

• Ubc9-overexpressing NSCs demonstrate enhanced neuronal differentiation.
• Upregulating SUMOylation in NSCs increases resistance to ischemia/reperfusion *in vitro.*
• Ubc9-overexpressing NSC grafts robustly integrate within the brain of mice post-stroke.

## Graphical abstract

**Figure:**
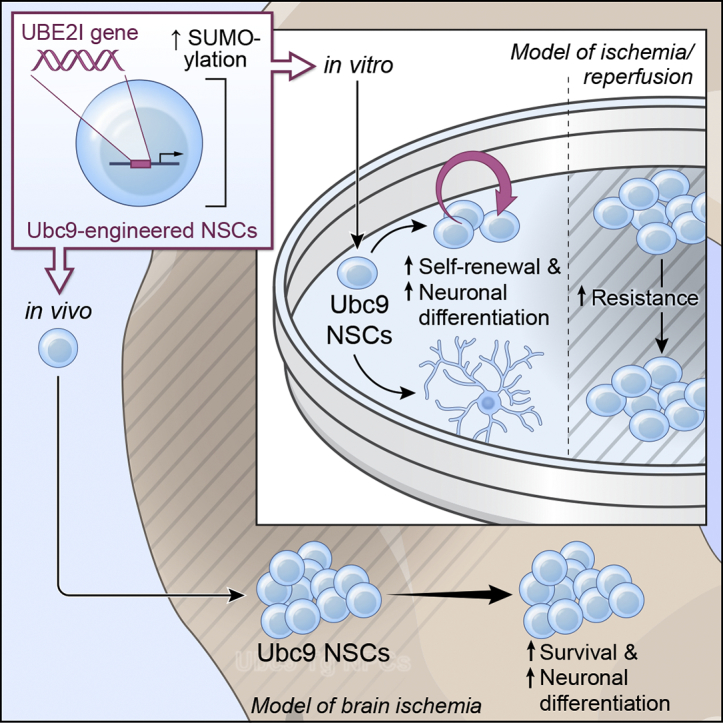

**Research in context**

**Evidence before this study**

Several studies have demonstrated the preclinical efficacy of NSC therapies with regard to their ability to protect and/or repair the ischemic brain. Transplanted NSCs exert beneficial effects not only *via* the structural replacement of dysfunctional and/or damaged endogenous cells, but also *via* immunomodulatory and neurotrophic actions. Unfortunately, the successful translation of such promising cell-based approaches remains elusive, in part due to the fact that grafted NSCs often die in the hostile ischemic/post-ischemic microenvironment.

**Added value of this study**

Ischemic stroke continues to pose a massive burden of disease and is a leading cause of morbidity and mortality throughout the world. However, beyond thrombolysis/mechanical thrombectomy we possess few effective therapies that are able to modulate the pathogenesis of evolving ischemic brain injury. The paucity of therapeutic options stands in stark contrast to the intensity of research efforts/number of clinical trials that have been performed to date. Further, there are, as of yet, no effective treatments that improve functional recovery in post-ischemic patients (*i.e.* regenerative therapies).

**Implications of all available evidence**

While NSC-based therapy for stroke holds great promise fundamental questions/problems persist and in so doing hinder the clinical translation of such approaches. As such, the work presented herein sought to explore the effects of the recruitment and optimization of a global neuroprotective modality (*i.e.* SUMOylation) on the efficacy of NSC cell-based therapy. Such work may ultimately find utility not only in the realm of stroke therapy, but could also extend to a wide variety of other degenerative/inflammatory neurological disorders that share components of stroke pathobiology (*e.g.* Alzheimer's, multiple sclerosis, spinal cord injuries, *etc.*).

Alt-text: Unlabelled Box

## 1 Introduction

In ischemic stroke a crisis in energy availability due to the sudden reduction of oxygen and glucose leads to the depletion of cellular energy stores, aberrant neurotransmitter release, and intense cytotoxic/inflammatory responses [1]. The intrinsic complexity of this phenomena accounts for the continuing failure of clinical approaches targeting single pathogenic mechanisms. As such, there is currently no effective treatment that improves functional recovery and replaces lost neural tissue in stroke patients.

Compelling evidence exists that the transplantation of neural stem cells (NSCs) exerts beneficial effects in multiple CNS disorders, including stroke, through both the structural replacement of endogenous neural cells as well as immunomodulatory and tissue trophic effects [[2], [3], [4]]. However, the metabolic demands of cellular grafts are only partially met, due to poor perfusion of post-ischemic brain tissue [5]. Accordingly, very few transplanted NSCs survive and integrate in the hostile post-ischemic microenvironment, thus hindering the therapeutic potential and successful translation of NSC-based approaches in stroke patients [6]. Counteracting cell death, the predominant fate of the NSC graft, therefore provides a clear target for interventions that aim at increasing the integration of cellular grafts [7].

A candidate pathway involved in resistance to the ischemic stress is the post-translational modification of proteins by the Small Ubiquitin-like MOdifier (SUMO), a process called SUMOylation [[8], [9], [10]]. We first described a striking increase in levels of global SUMOylation during hibernation torpor in 13-lined ground squirrels (*Ictidomys tridecemlineatus*) [8,11]. These animals showed no evidence of cellular damage or functional deficits despite prolonged exposure to low perfusion levels similar to those observed within the “ischemic core” [11]. Of interest, recent work has even come to suggest a role for SUMOylation within the human penumbra [12].

Mechanistically, SUMOylation requires a cascade of enzymatic steps that involves an E1 activating enzyme, an E3 SUMO ligase, and the sole E2 conjugase Ubc9, which is coded by the *UBE2I* gene [9]. Transgenic mice overexpressing Ubc9 showed increased global SUMOylation, with levels of SUMOylation directly proportional to the degree of cytoprotection [13,14].

Herein, we sought to engage SUMOylation in an effort to engineer NSC grafts capable of surviving within unhospitable ischemic microenvironments. We established Ubc9-overexpressing NSCs and characterized their global gene/protein expression profiles, phenotype, and function, both *in vitro* and *in vivo*. The upregulation of neurogenic pathways in Ubc9 NSCs led to enhanced neuronal differentiation *in vitro.* Ubc9 NSCs also displayed a strikingly-increased resistance to oxygen-glucose deprivation and restoration of oxygen/glucose (OGD/ROG) *in vitro*. Finally, when transplanted directly into the ischemic lesion of mice with middle cerebral artery occlusion (MCAO), Ubc9 NSC grafts showed a significant, 2-fold higher level of survival and integration *in vivo* as compared to control NSC grafts.

Increasing SUMOylation in NSCs represents a novel approach to graft preconditioning that has the potential to overcome some of the current limitations of stem cell medicines within regenerative neurology.

## 2 Materials and methods

### 2.1 Contact for reagent and resource sharing

Further information and requests for resources and reagents should be directed to and will be fulfilled by the Lead Contacts: Joshua D. Bernstock (bernstockjd@ninds.nih.gov); Luca Peruzzotti-Jametti (lp429@cam.ac.uk); John M. Hallenbeck (hallenbj@ninds.nih.gov); and Stefano Pluchino (spp24@cam.ac.uk).

### 2.2 Intraluminal (temporary) middle cerebral artery occlusion (MCAO), and stereotaxic NSC transplantation

Adult male C57NL/6 mice (10–12 weeks old) were purchased from Charles River. Animals were anesthetized with 1–1.5% isoflurane. Temperature was maintained between 36.5 °C and 37.0 °C, and laser Doppler flow was monitored during the procedure and for up to 15 min post-reperfusion to ensure the return of cerebral blood flow (CBF). Focal cerebral ischemia of the left MCA was induced with a silicon-coated 6–0 nylon filament (Doccol Corporation). The filament was retracted after 45 min allowing reperfusion. At 72 h after ischemia, animals were randomized to receive intraparenchymal transplantation of either GFP-labelled WT NSCs or GFP-labelled Ubc9 NSCs (both at passage ≤15). A 26S gauge small hub RN needle 2 in point style 3 (Hamilton Company, Reno, NV) was inserted at the following coordinates, with the origin at bregma: anterior-posterior [+] 0.5 mm, *mediolateral [−] 1.8 mm,* dorsoventral [−] 2.6 mm. A 0.5 mm reservoir was created using the needle immediately prior to cell injection. A total of 100,000 cells in a total volume of 2 μl was injected slowly and continuously over a period of 5 min. Post-injection, the needle was withdrawn continuously at a rate of 0.1 mm per 30 s and the surgical incision was sutured. The equipment used in the transplantation procedure is as follows: 5 μl Model 75 RN syringe (Hamilton Company), Benchmark Stereotaxic Frame (Leica Biosystems), Benchmark Digital Stereotaxic Control Panel (Leica Biosystems). All animal experiments conformed to the guidelines set forth by the NIH/NINDS Animal Care and Use Committees (ACUC) (protocol #1268-15).

### 2.3 Genotyping of transgenic animals and cell lines

Transgenic animals and derived cell lines were genotyped *via* touchdown PCR to confirm the presence of the *UBE2I* transgene. Part of the CAG promoter sequence (5′-gcgccggcaggaaggaaatg-3′) was used as a forward primer and part of the Ubc9 coding sequence (5′-ggtgatagctggcctccagtcc-3′) was used as a reverse primer. Only the transgene (*CAG-UBE2I*) is amplified as a 650-base pair DNA fragment. SHSY5Y cell extracts (total DNA preps) from trans gene inserted plasmid are used as positive control.

### 2.4 NSC derivation and cell culture

Adult neurospheres were generated from the SVZ of both WT C57BL/6 and Ubc9 transgenic 4–8-month-old mice. Briefly, mice were anesthetized by an intraperitoneal injection of pentobarbital and sacrificed *via* cervical dislocation. The brains were removed and placed in artificial cerebrospinal fluid (aCSF) (124 mM NaCl, 5 mM KCl, 1.3 mM MgCl_2_, 0.1 mM CaCl_2_, 26 mM NaHCO_3_, and 10 mM d-glucose, pH 7.4) aerated with 95%, O_2_, 5% CO_2_ at room temperature. SVZ neural tissue was isolated after coronal sectioning and cut into 1 mm^3^ pieces. Pieces were transferred into 30 ml of aCSF containing 1.3 mg/ml trypsin, 0.67 mg/ml hyaluronidase, and 0.2 mg/ml kynurenic acid (Sigma-Aldrich) and incubated, under continuous oxygenation and stirring, for 90 min at 32–34 °C. Tissue sections were then rinsed in aCSF for 10 min, transferred to DMEM/F12 (Thermo Fisher Scientific) medium containing 0.7 mg/ml ovomucoid (Sigma-Aldrich), and carefully triturated using a fire-polished Pasteur pipette. Cells were collected by centrifugation and resuspended in growth factor-free, chemically-defined DMEM/F12 medium containing 2 mM l-glutamine, 0.6% glucose, 9.6 mg/ml putrescine, 6.3 ng/ml progesterone, 5.2 ng/ml sodium selenite, 0.025 mg/ml insulin, 0.1 mg/ml transferrin, and 2 mg/ml heparin (Sigma-Aldrich). Cells were then cultured in NS-A medium (Euroclone) containing 20 ng/ml of epidermal growth factor (EGF) and 10 ng/ml fibroblast growth factor (FGF)-II (Peprotech). The number of primary spheres was counted after 7–12 days *in vitro*. For cell amplification, 8000 cells/cm^2^ were plated at each sub-culturing passage in untreated tissue culture flasks. NSC Proliferation Medium which consisted of 10% NeuroCult Proliferation Supplement (Stem Cell Technologies), 1% Pen/Strep, 0.1% *v*/v Heparin (Sigma), 0.01% v/v EGF (Peprotech), 0.01% v/v bFGF (Peprotech) in NeuroCult Basal Medium (Stem Cell Technologies) was used for growth/cell maintenance and incubated at 37 °C and 5% CO_2_. After ~ 4 days neurospheres were harvested, enzymatically dissociated using Accumax (Stem Cell Technologies) counted using trypan blue and ultimately re-plated under the same culture conditions.

### 2.5 NSC growth curves, viability, clonogenicity and cell cycle *in vitro*

The linear growth curve was generated by calculating the total number of cells by multiplying the growth rate (*i.e.* number of live cells divided by the number of seeded cells) by the total number of cells present at the previous time point. The mean of the total number of cells per time point ± standard error of the mean (SEM) was reported to build the linear trend line. The daily growth rate was obtained by dividing the growth rate by the number of days per passage. Viability was defined as the percentage of viable cells over dead cells ±SEM. For clonogenicity assays, single cells were sorted *via* flow cytometry into 100 μL of NSC proliferation media/well in flat bottom 96-well plates. 7 days later the number of neurospheres were counted and scored (*i.e.* size and total number). For cell cycle analysis, 350,000 WT or Ubc9 NSCs per well were plated into 6-well plates pre-coated with Matrigel Matrix (Corning). After the incubation period, cells were fixed, permeabilized, and stained using the Click-iT EdU Plus Flow Cytometry Assay Kit (ThermoFisher Scientific) as per the manufacturer's instructions. This was followed by counterstaining with 1 μg/ml 4′,6-diamidino-2-phenylindole (DAPI) to assay total DNA content per cell. Cells were then analysed by flow cytometry using a MoFlo Astrios cell sorter with Summit Acquisition software (Beckman Coulter); 5 × 10^4^ events were acquired per sample. Data analysis was completed with Kaluza software (Beckman Coulter).

### 2.6 NSCs differentiation *in vitro*

For differentiation assays *in vitro*, cells were seeded on 13 mm glass coverslips pre-coated with Matrigel™ (Corning) at a density of 8 × 10^4^ cells/coverslip and cultured in 400 μl differentiation medium (NeuroCult™ basal medium) (Stem Cells Technologies) with 10% mouse differentiation supplement (Stem Cells Technologies), 1% pen/strep]. Half of the medium was replaced with fresh differentiation medium after 3 days. After 3 more days (6 days in total), coverslips were washed with PBS and fixed with 4% paraformaldehyde (Sigma-Aldrich) and 2% sucrose in PBS. For immunofluorescence staining, cells were rinsed with PBS, and then blocked for 1 h at room temperature in blocking buffer (0.1% Triton X100 and 10% secondary antibody species serum in PBS). Primary antibodies were incubated at 4 °C overnight. The following primary antibodies diluted in blocking buffer were used: anti-nestin (1:200) (Abcam), anti-SOX2 (1:100) (Abcam), anti-vimentin (1:100) (Abcam), anti-glial fibrillary acidic protein (GFAP) (1:500) (Abcam), anti-β-tubulin-III (1:500) (BioLegend), anti-O4 (1:400) (R&D Systems). Cells were then washed in PBS with 0.1% Triton X100 and incubated with the appropriate fluorescent secondary antibodies (1:1000 Alexa-Fluor 405, 488, 555, 647, Invitrogen) 1 h at room temperature. After washing in PBS, nuclei were counterstained with 1 μg/ml DAPI (Invitrogen) for 3 min and then mounted with Dako mounting kit (Sigma-Aldrich). Nonspecific staining was observed in control incubations in which the primary antibodies were omitted. For quantification, images were acquired using a CCD camera (DC 480) (Leica) equipped fluorescence microscope (Olympus BX51) (Olympus) with a 40× objective on 6 regions of interest (ROI) per coverslip. Images were analysed and prepared using ImageJ software (NIH, Bethesda, MD). Data are presented as the percentage of positive cells over the total of DAPI positive cells ± SEM.

### 2.7 XF assays

Oxygen consumption rate (OCR) and extracellular acidification rate (ECAR) were measured using the Seahorse XF24 Extracellular Flux Analyser (Seahorse Bioscience). Undifferentiated WT and Ubc9 NSCs were dissociated and seeded into a 24 well XF24^e^ cell culture microplate (3.3 × 10^5^ cells/cm^2^) pre-coated with Matrigel™ (Corning) at a final density of 1 × 10^5^ cells/well in normal media. The assays were performed 18 h after seeding. For differentiated WT and Ubc9 NSCs we adapted the protocol described above (*NSCs differentiation in vitro*) to the XF24^e^ cell culture microplate. Briefly, undifferentiated NSCs were seeded after dissociation on a 24 well XF24^e^ cell culture microplate pre-coated with Matrigel™ (Corning) at a final density of 3.3 × 10^5^ cells/cm^2^ and cultured in 400 μl differentiation medium [NeuroCult™ basal medium (Stem cells Technologies), 10% mouse differentiation supplement (Stem Cells Technologies), 1% pen/strep]. 200 μl of differentiation medium were replaced with fresh medium after 3 days. Differentiation was continued until day 6, and assays were performed the following day. Before XF analysis, culture media were removed, cells were washed with sterile PBS, and XF medium pH 7.35–7.45 was added and allowed to equilibrate for 30 min at 37 °C. Data were normalized on total protein content per well evaluated using a BCA assay kit (Thermo Fisher Scientific) and are expressed as pmoles/min (OCR) or milli-pH units (mpH)/min (ECAR) mean ± SEM.

### 2.8 Oxygen/glucose deprivation (OGD)/restoration of oxygen/glucose (ROG) and FACS analysis of live/dead cells

We subjected NSCs (both differentiated and undifferentiated) to OGD for 5 h followed by ROG for 18 h. RNA for the microarrays was harvested 1 h into reperfusion. To induce OGD, the basal culture medium was replaced with a glucose-free, balanced salt solution (BSS), which contained (in mmol/L) the following: NaCl, 116; CaCl_2_, 1.8; MgSO_4_, 0.8; KCl, 5.4; NaH_2_PO_4_, 1; NaHCO_3_, 14.7, and HEPES, 10 (pH 7.4). Cell death was assessed *via* nuclear staining with Hoechst 33342 and propidium iodide (PI) followed by FACS analysis. Typically, 1 × 10^5^ cells were analysed. The percentages of vital or apoptotic/necrotic cells in the OGD condition were normalized to their corresponding non-OGD sample to control for baseline death rate before deriving the fold difference *vs* the WT average.

### 2.9 Cleaved caspase-3 activity

Apoptosis in undifferentiated WT and Ubc9 NSCs after OGD/ROG was also assessed with an assay for cleaved caspase-3. Cells were collected and analysed according to the manufacturer's instructions (R&D Systems). Cleaved caspase-3 levels in Ubc9 NSCs were normalized as fold differences relative to their corresponding passage-matched WT.

### 2.10 Immunoprecipitation (IP) and western blot analyses

For the global SUMO-1 pulldown, after removal of the media and washing with phosphate buffered saline (PBS), cells were lysed in an IP buffer (50 mM HEPES pH 7.3, 100 mM NaCl, 1.5 mM MgCl_2_, 1% NP40, 0.1% SDS, 1 mM PMSF, 20 mM NEM, MS-SAFE protease/phosphatase inhibitor cocktail). The cell lysates were then incubated for 1 h on ice and then centrifuged for 20 min at ~12,000 x g at 4 °C. After pre-clearing with Dynabeads Protein G (Thermo Fisher Scientific), protein concentrations were measured with a Pierce BCA Protein Assay (Thermo Fisher Scientific). Equal amounts of protein among the relevant samples were ultimately used for IP. After incubation with the primary antibody for 2 h at 4 °C, Dynabeads Protein G were added and incubated overnight at 4 °C. The washing and eluting of IP products that followed conformed entirely to the manufacturer's (Thermo Fisher Scientific) protocol. Total cell lysates for western blots from NSCs were prepared and the antibodies used throughout the course of this study were as follows: rabbit polyclonal anti-SUMO-1 and anti-SUMO-2/3 antibodies (both developed in-house), rabbit monoclonal anti-UBE2I/Ubc9 (Abcam), and anti-β-actin (Sigma-Aldrich). Protein expression levels were determined *via* densitometric analysis of the corresponding protein bands of interest using ImageJ (NIH, Bethesda, MD). To measure SUMO-conjugation levels, regions corresponding to molecular weights above 100 kDa in each lane were cropped and the total intensity analysed. All densities were normalized to the corresponding actin levels and expressed as a fold difference *vs* WT NSCs.

For the Sox2 IP, after removal of the media and washing with phosphate buffered saline (PBS), cells were lysed in Pierce IP Lysis Buffer (Thermo-Fisher Cat: 87788) with Halt Protease and Phosphatase Inhibitor Cocktail (Thermo-Fisher Cat: 78440). IP was performed with Dynabeads Protein G (Thermo-Fisher Cat: 10003D) conjugated with a mouse anti-Sox2 primary antibody (Abcam Cat: 171380), following manufacturer's instructions. Samples were then loaded on Mini-PROTEAN TGX Stain-Free Gels (Biorad Cat: 456–8086), transferred and probed with the mouse anti-Sox2 primary antibody (Abcam Cat: 171380), a rabbit anti-SUMO-1 primary antibody (Cell Signaling Technology Cat: 4930) and relevant anti mouse/rabbit HRP secondary antibodies (GE Health Care Cat: NXA931and Thermo-Fisher Cat: 31402 respectively).

### 2.11 Proteomic identification & pathway enrichment analysis

Protein identification of excised Coomassie Blue-stained gel bands was performed by in-gel tryptic digestion, analysis of the resulting digest by liquid chromatography coupled to mass spectrometry LC/MS, and database searching. Briefly, samples were digested with trypsin overnight at 37 °C. Tryptic peptides were desalted first and analysed on an Orbitrap Elite mass spectrometer (Thermo Scientific). Peptides were separated on an Ultimate 3000 HPLC (Thermo-Dionex) using a ES802 Easy-Spray column (75-μm inner diameter, 25 cm length, 3 μm C_18_ beads; Thermo Scientific). A 55-min linear gradient of 2–27% mobile phase B (mobile phase A: 2% acetonitrile, 0.1% formic acid; mobile phase B: 98% acetonitrile, 0.1% formic acid) was used. The Thermo Scientific Orbitrap Elite mass spectrometer was operated in positive nano-electrospray mode. The CID MS/MS data were acquired in data dependent mode. The top ten most abundant ions were selected for product ion analysis. Mass spectrometry-identified proteins for two separate SUMO1 pull-down fractions representing the WT NSC condition and the Ubc9 NSC condition were filtered to retain proteins having at least 2 significant peptide matches in at least one fraction representing WT NSC condition and/or in at least one fraction representing the Ubc9 NSC condition. For these proteins, three lists were organized by symbol: detected in both WT NSC and Ubc9 NSC conditions, detected in WT NSC condition only, and detected in Ubc9 NSC condition only. For proteins detected in the Ubc9 NSC condition only, symbols were import into IPA (www.ingenuity.com) and the corresponding enriched functions and pathways identified.

### 2.12 Microarray analysis (Affymetrix *Mouse* Gene *2.0 ST Arrays)*

Total RNA was extracted by samples using the RNeasy Midi Kit (QIAGEN) then labelled according to the manufacturer's guidelines for use with the Mouse Gene 2.0 Array (Affymetrix). Labelled cRNA were hybridized to these arrays in a blinded interleaved fashion. The Scanner 3000 (Affymetrix) was used in conjunction with the GeneChip Operation Software (Affymetrix) to generate one CEL file per hybridized cRNA. The CEL files were then loaded into R, RMA was normalized with the *oligo* package, and filtered to only retain probes annotated as “main”. Differential expression testing was performed using *limma* and the resulting p.values were corrected with the Benjamini-Hochberg method. To test the interaction between OGD/ROG response and genotype, we added the following contrasts to the contrasts.fit function of limma: (Ubc9_NDiff_OGDROG-Ubc9_NDiff_CNTL)-(NPC_Ndiff_OGDROG-NPC_Ndiff_CNTL) and (Ubc9_Diff_OGDROG-Ubc9_Diff_CNTL)-(NPC_diff_OGDROG-NPC_diff_CNTL). GO enrichment analyses were performed using the topGO package (https://bioconductor.org/packages/release/bioc/html/topGO.html) with the *classic* algorithm and *Kolmogorov-Smirnov* statistic. Clustering for microarray heatmaps was realized using Euclidean distance and the hclust algorithm (complete method). The KEGG pathway enrichment analysis were performed with the CAGE packages using lists of genes sorted by log2 fold change.

### 2.13 Lentiviral GFP tagging

Cells used for transplantation studies were transduced *in vitro* using a third-generation lentiviral carrier (pRRLsinPPT-hCMV) coding for the enhanced farnesylated green fluorescent protein (GFP), which targets the fluorescent protein to the inner plasma membrane of transduced cells. Neurospheres were harvested, dissociated to a single cell suspension, and seeded at high density [1.5 × 10^6^ in a T75 cm^2^ flask (Sigma-Aldrich)] in fresh medium. After 12 h, 3 × 10^6^ T.U./ml of lentiviral vectors were added. 6 h later, fresh medium was added. 72 h after viral transduction, cells were harvested and re-seeded at a normal concentration. Transgene expression was measured by FACS analysis before transplantation and > 98% of cells were found to be labelled in both lines (*data not shown*).

### 2.14 Ex vivo pathology and stereological analysis

Five- or 30-days post-transplantation (dpt) of WT and Ubc9 NSCs, mice were deeply anesthetized with isoflurane and transcardially perfused with PBS for 15 min, followed by a solution of 4% PFA in PBS for 15 min. Brains were then exposed, isolated, post-fixed overnight in 4% PFA and ultimately cryopreserved in 10–30% sucrose in PBS at 4 °C for 24–48 h. Brains were then embedded in optimum cutting temperature (OCT) medium, frozen using liquid nitrogen, cryo-sectioned coronally at a thickness of 20 μm and stored at −80 °C. Quantification of total ischemic volume was performed using cresyl violet (CV) stained brain sections. Briefly, sections were first dehydrated with increasing dilutions of ethanol in water, washed in xylene (Merck), and rehydrated with decreasing dilutions of ethanol in water. Sections were then incubated with the CV solution for 10 min at room temperature. Stained tissues were then dehydrated with 75%, 95% and 100% ethanol diluted in water, washed in xylene, and mounted using a synthetic mounting medium (EUKITT). Data are expressed as percentage of contralateral hemisphere volume ± SEM of *n* = 12 sections per mouse. For quantification of WT and Ubc9 NSC graft survival, 20 μm coronal sections were pre-treated with peroxidase 3% for 15 min, incubated in a blocking solution which consisted of PBS + 10% normal goat serum (NGS, Sigma-Aldrich) for 1 h at room temperature, and then incubated with chicken anti-GFP (1:500) diluted in PBS and 1% NGS at 4 °C overnight. Sections were washed with PBS and incubated with goat anti-chicken biotinylated secondary antibody (1:500, Sigma-Aldrich) diluted in PBS and 1% NGS for 1 h at room temperature. Components “A” and “B” of the Vectastain kit (Vector Laboratories) were pre-mixed for 45 min and sections were incubated with this solution for 1 h at room temperature. The stain was developed by means of 3,3′-diaminobenzidine (DAB) following the manufacturer instructions. After 30 s the reaction was stopped by dipping the section into distilled water. Sections were then counterstained with haematoxylin for 30 s at room temperature and mounted using Dako mounting medium (Fluka). The mean thickness of analysed sections was 17.64 μm (shrinkage to 88.2% of the original thickness). Transplanted GFP^+^ cells were quantified according to the optical fractionator method on *n* = 12 equally spaced sections with the assistance of an Olympus BX53 microscope with motorized stage and Neurolucida software (11.07 64-bit, Microbrightfield); the motorized stage of the microscope controlled by the software allowed precise and well-defined movements along the x-, y- and z-axes. The counting was performed using a 100× oil immersion lens. Data are expressed as number of counted cells ± SEM.

For the detection of apoptotic cells within coronal brain sections a Click-iT™ Plus TUNEL Assay for *in situ* apoptosis detection (Alexa Fluor™, ThermoFisher) was used in combination with primary antibodies (Ki67 at 1:500) as per the manufacturer's instructions. Briefly, after washing, sections were incubated for 1 h at room temperature with the appropriated combinations of secondary antibodies (1:1000 Alexa-Fluor 488, 555, 647, Invitrogen). Nuclei were counterstained with DAPI (1:10,000, Invitrogen) for 3 min at room temperature. For quantification of NSC differentiation at 30 dpt, 20 μm coronal brain sections were washed with PBS and blocked for 1 h at room temperature in 0.3% Triton X100 and 10% secondary antibody species serum in PBS. A Fab fragment affinity-purified IgG anti-mouse was used if anti-mouse antibody were incubated with sections (1:10, Jackson ImmunoResearch). The following primary antibodies were then incubated overnight at 4 °C: anti-GFP (1:250) (Invitrogen), anti-GFAP (1:500) (Abcam), anti-NeuN (1:200) (Chemicon), anti-Olig2 (1:500) (Millipore). Differentiation data are expressed as % GFP^+^ cells that are reactive for a specific marker.

To determine the percentage of WT and Ubc9 NSCs that have differentiated into neurons and to study the synapse formation of these neurons, cryo-sections of the brain were stained for markers specific for microtubule-associated protein 2 (anti-MAP2) (1:200) (Abcam) and for postsynaptic density protein 95 (anti-PSD95) (1:300) (Abcam) while concurrently staining for the GFP (1:200) (Invitrogen) labelled NSCs. Sections were briefly rinsed in distilled water and washed with PBS. After blocking of the sections in blocking buffer (PBS + 0.1% Triton X100) with 10% NGS for 1 h at room temperature, and blocking with 1:10 goat anti-mouse IgG (in blocking buffer, the primary antibodies were diluted in blocking buffer with 10% NGS and incubated overnight at 4 °C. Sections were then washed, incubated with the appropriate secondary antibodies (1:400 Alexa-Fluor 488, 546, 647, Invitrogen) for 2 h at 4 °C, and washed again. Nuclei were counterstained with DAPI (1:10,000) (Invitrogen) for 3 min at room temperature. Sections were washed and mounted using Dako fluorescence mounting medium (Agilent). Images of the staining were acquired with an Andor dragonfly confocal microscope (Oxford Instruments) and analysed using Fiji software. Data is expressed as % GFP^+^ area that is positive for MAP2 or PSD95.

### 2.15 Quantification and statistical analysis

Data are mean values ± SEM. The 2-tailed Student's *t*-test was used for single comparisons. Protein mass spectrometry data were analysed with Fisher's Exact Test. *p*-value<.05 was considered significant. Gene expression profiling assays were realized with Mouse Gene 2.0 Arrays (Affymetrix). See specific sections for the detailed experimental and analytical procedures.

### 2.16 Data and software availability

The microarray data have been deposited on GEO with the accession number E-MTAB-7428.

## 3 Results

### 3.1 Overexpression of Ubc9 in NSCs induces genes and proteins governing cell cycle and neurogenesis *in vitro*

**Fig. 1:**
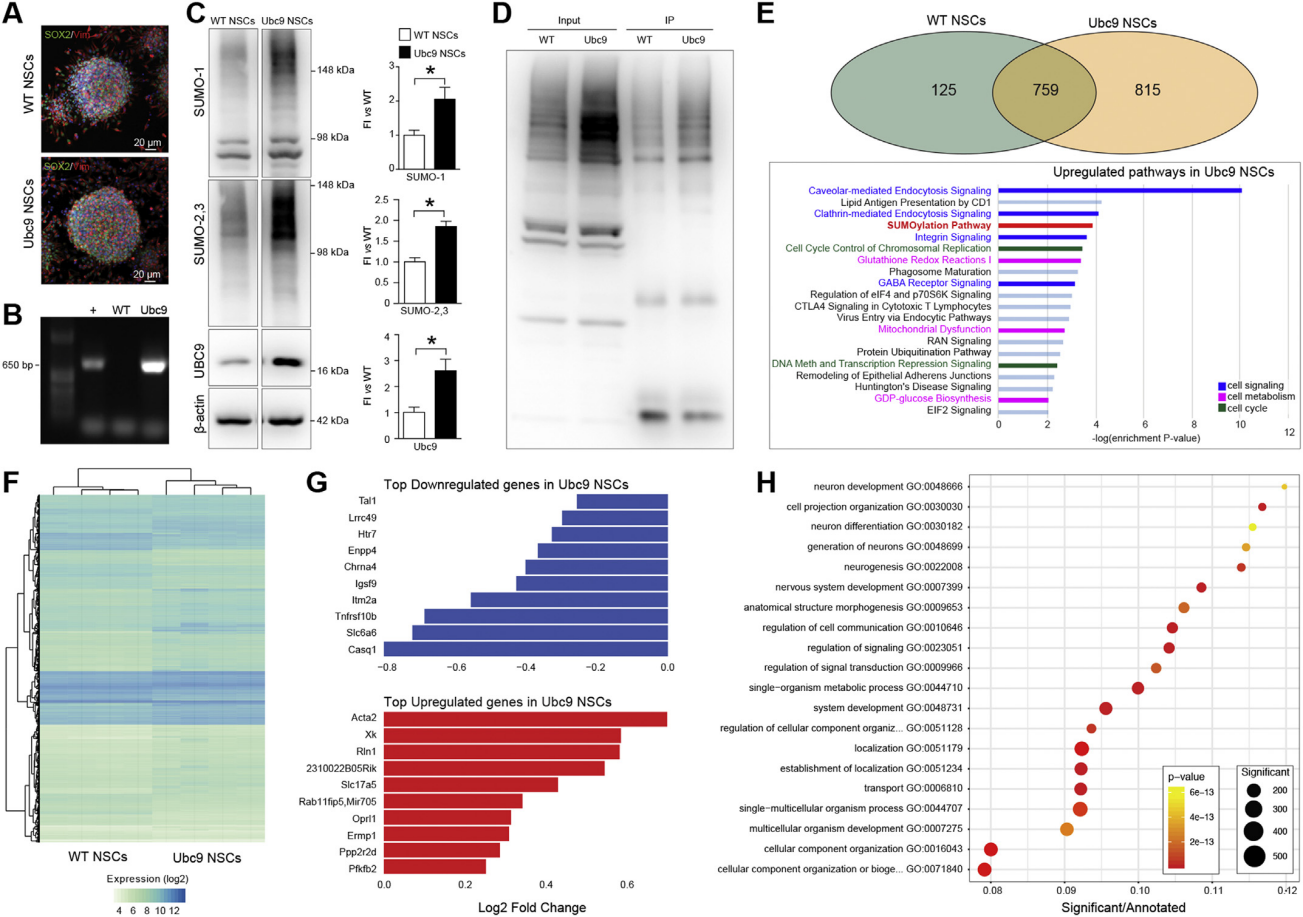
Overexpression of Ubc9 induces genes and proteins related to cell signaling, cell metabolism and cell cycle. (A) Representative confocal images of WT NSCs and Ubc9 NSCs growing *in vitro* as SOX2^+^/Vimentin (Vim)^+^ neurospheres. Nuclei are stained with DAPI. See also Fig. S1. (B) Genomic PCR for *CAG-UBE2I* transgene in Ubc9 NSCs (+: positive control). (C) Representative immunoblots and densitometries normalized to corresponding actin levels and expressed as a fold induction (FI) relative to WT NSCs. Data are means ± SEM. *N* ≥ 3 per group, * *p*-value<0.05 (2-tailed Student's *t*-test). (D) Global SUMO-1 pulldown in WT NSCs and Ubc9 NSCs. Input = precleared lysate, IP = immunoprecipitated lysate. See also Data S1. (E) Venn diagram of protein mass spectrometry data showing that 125 proteins co-immunoprecipitated with SUMO-1 in WT NSCs only, 815 proteins in Ubc9 NSCs only, and 759 were common to both. Pathways and functions significantly upregulated in Ubc9 NSCs compared to WT NSCs based on protein mass spectrometry results are reported. *N* = 2 per group (Fisher's Exact Test). See also Data S1 and Fig. S1. (F) Heatmap of genes differentially expressed in Ubc9 NSCs *vs* WT NSCs. *N* = 4 per group, adjusted p-value<0.05. See also Data S2. (G) Bar chart of the fold change (log2) in Ubc9 NSCs *vs* WT NSCs of the 10 most upregulated and downregulated genes. Adjusted p-value<0.05. See also Data S2. (H) Plot of the GO enrichment results for genes differentially expressed in Ubc9 NSCs *vs* WT NSCs. The x-axis shows the enrichment ratio, the color indicates the enrichment p-value (Kolmogorov–Smirnov statistic) and the size of each point indicates the number of significant genes in the corresponding GO category. See also Data S2.

We established NSC lines from the subventricular zones (SVZ) of adult Ubc9-overexpressing mice (Ubc9 NSCs) [13] and control wild-type mice (WT NSCs) (Fig. 1A and B).

We found that global SUMO-1 and SUMO-2/3 conjugation, as well as Ubc9 protein levels, were increased in Ubc9 NSCs (Fig. 1C). We then performed global SUMO-1 immunoprecipitation (IP) (Fig. 1D) and protein pathway analysis (Fig. 1E) confirming that, beyond SUMOylation and ubiquitination, protein pathways related to cell signaling, cell metabolism, and cell cycle were prevalent in Ubc9 NSCs (Data S1). Ubc9 NSCs also displayed significantly higher growth rates and self-renewal *in vitro*, with an increased proportion of cells in the S-phase of the cell cycle (Fig. S1).

We next performed a microarray expression analysis of Ubc9 NSCs (Fig. 1F and Data S2) and found that 497 and 922 genes were significantly downregulated and upregulated, respectively, in Ubc9 NSCs (*p*-value<0.05).

On one hand, Ubc9 NSCs demonstrated a significant reduction in the expression levels of genes related to apoptosis, including the death receptor *Tumor Necrosis Factor Receptor Superfamily* (*Tnfrsf*)*10b*, the regulator of autophagy *Integral Membrane Protein 2A* (*Itm2a*), and the *cholinergic receptor nicotinic alpha 4 subunit* (*Chrna4*) (Fig. 1G).

On the other hand, the cell cycle regulator *Protein Phosphatase 2 Regulatory Subunit B delta* (*Ppp2r2d*) [15], and the regulator of glycolytic metabolism *6-phosphofructo-2-kinase/fructose-2,6-biphosphatase 2 (Pfkfb2*) [16], were significantly upregulated in Ubc9 NSCs (Fig. 1G). Notably, the majority of the upregulated genes in Ubc9 NSCs were related to enhanced neurogenesis and neuronal function [*e.g. actin alpha 2 smooth muscle aorta* (*Acta2*); the regulator of neuronal intracellular trafficking *Kx Antigen* (*Xk*), the hormone *Relaxin-1* (*Rln-1*) and the sialic acid transporter *Solute Carrier* (*Slc*) *17a5*] (Fig. 1G).

Gene Ontology (GO) enrichment analysis of the expression data showed that the six most enriched pathways affected by the Ubc9 transgene were related to neural development, function, or differentiation (Fig. 1H).

These data suggest that the presence of the Ubc9 transgene in NSCs not only increases global SUMOylation levels, cellular growth rates, and self-renewal *in vitro*, but also has effects on gene expression, reducing transcripts related to apoptosis and increasing the expression of neurogenic genes.

### 3.2 Ubc9 NSCs display a quiescent metabolic profile upon differentiation and are predisposed to neuronal differentiation in vitro

**Fig. 2:**
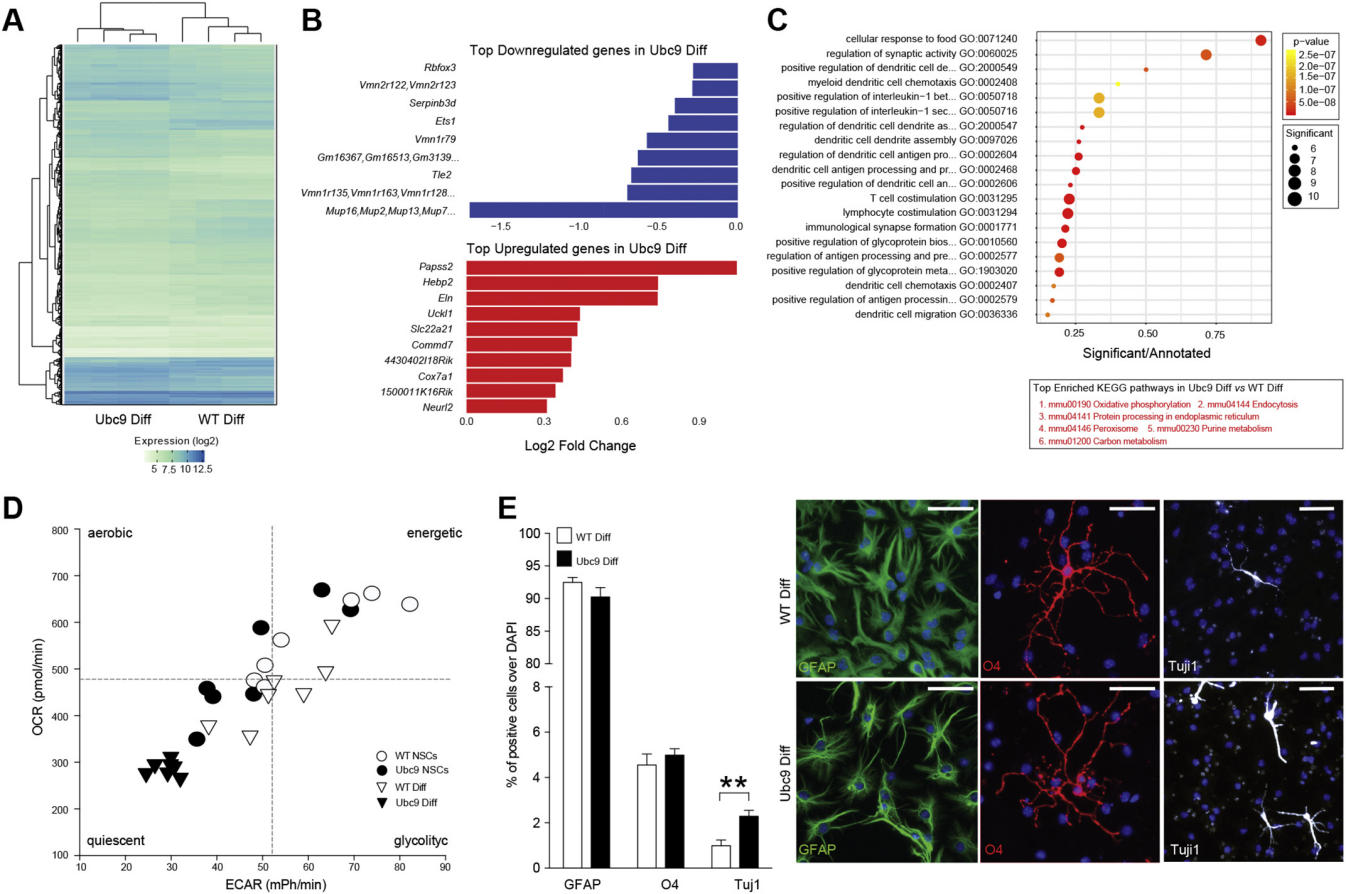
Ubc9 NSCs show a quiescent metabolic profile upon differentiation and are predisposed to neuronal differentiation *in vitro.* (A) Heatmap of genes differentially expressed in Ubc9 Diff *vs* WT Diff cells. N = 4 per group, adjusted *p*-value<0.05. See also Data S2. (B) Bar chart of the fold change (log2) in Ubc9 Diff *vs* WT Diff cells of the 10 most upregulated and downregulated genes. All genes have an adjusted p-value<0.05. See also Data S2. (C) Plot of the GO enrichment results for genes differentially expressed in Ubc9 Diff *vs* WT Diff cells. The x-axis shows the enrichment ratio, the color indicates the enrichment p-value (Kolmogorov–Smirnov statistic), and the size of each point indicates the number of significant genes in the corresponding GO category. The table at the bottom shows the 6 KEGG pathways showing the strongest enrichment (GAGE analysis p-value<0.05 for all pathways, see methods) in the same comparison. See also Data S2. (D) OCR *vs* ECAR graph of undifferentiated and differentiated WT and Ubc9 NSCs. Data are expressed as mean pmoles/min (OCR) and mean milli-pH units (mpH)/min (ECAR) ± SEM. *N* = 7 per group from 2 independent experiments. (E) Quantification and representative immunofluorescence-staining of WT Diff and Ubc9 Diff NSCs. Data are means ± SEM. *N* ≥ 6 per group from 2 independent experiments, **p-value<0.01 (2-tailed Student's *t*-test). Nuclei are stained with DAPI, scale bars: 50 μm.

To study the role of Ubc9 overexpression in the differentiation of NSCs, we cultured NSCs in the absence of epidermal growth factor (EGF) and fibroblast growth factor-2 (FGF-2) and examined their transcriptional profiles (Fig. 2A and Data S2).

Several genes were significantly downregulated in differentiated Ubc9 NSCs (Ubc9 Diff), including genes with known oncogenic potential [*e.g.* the *Serpin Family B Member 3* (*Srpinb3d*), the *ETS Proto-Oncogene 1* (*Ets1*), and the *Transducin-like enhancer protein 2* (*Tle2*)] and genes playing a role in olfactory neuron signaling [*vomeronasal receptors* (*Vmnr*) and *major urinary proteins* (*Mup*)] (Fig. 2B).

Conversely, we found that genes primarily involved in cell metabolism were upregulated in Ubc9 Diff, including the high affinity carnitine transporter *Slc22a21*, the *Cytochrome C Oxidase Subunit 7A1 (Cox7a1)* and the *Heme Binding Protein 2* (*Hebp2*) (Fig. 2B). A GO enrichment and Kyoto Encyclopedia of Genes and Genomes (KEGG) pathway enrichment analysis confirmed that metabolic pathways were most-highly enriched in Ubc9 Diff (Fig. 2C).

When we analysed the metabolic profile of Ubc9 Diff, we observed a remarkably low oxygen consumption rate (OCR) and extracellular acidification rate (ECAR) (Fig. 2D), which were both suggestive of quiescent metabolism.

Phenotypically, spontaneous *in vitro* differentiation of Ubc9 NSCs yielded similar fractions of glial fibrillary acid protein (GFAP)^+^ and O4^+^ glia compared to WT Diff. However, the fraction of Tuj1^+^ neurons was significantly higher (2.3-fold increase) in Ubc9 Diff (Fig. 2E).

Critically, we demonstrate that Sox2 – a key transcription factor essential for maintaining self-renewal of stem cells - is present in NSCs and that SUMOylation of Sox2 is upregulated in Ubc9 NSCs, compared to wild-type control NSCs (Fig. S2). As such, its inhibited function *via* SUMOylation might account for the enhanced neuronal phenotypes seen in Ubc9 NSCs *in vitro* [17].

Thus, upon differentiation *in vitro*, Ubc9 NSCs display increased neuronal differentiation with changes in genes related to cell metabolism, which may underlie the homeostatic metabolic profile of these cells.

### 3.3 Ubc9 overexpression induces gene expression changes that protect NSCs against OGD/ROG *in vitro*

To determine if any of the biological differences induced by the Ubc9 transgene would result in enhanced survival within the ischemic microenvironment, we exposed NSCs to OGD/ROG, and analysed gene expression as well as functional responses.

**Fig. 3:**
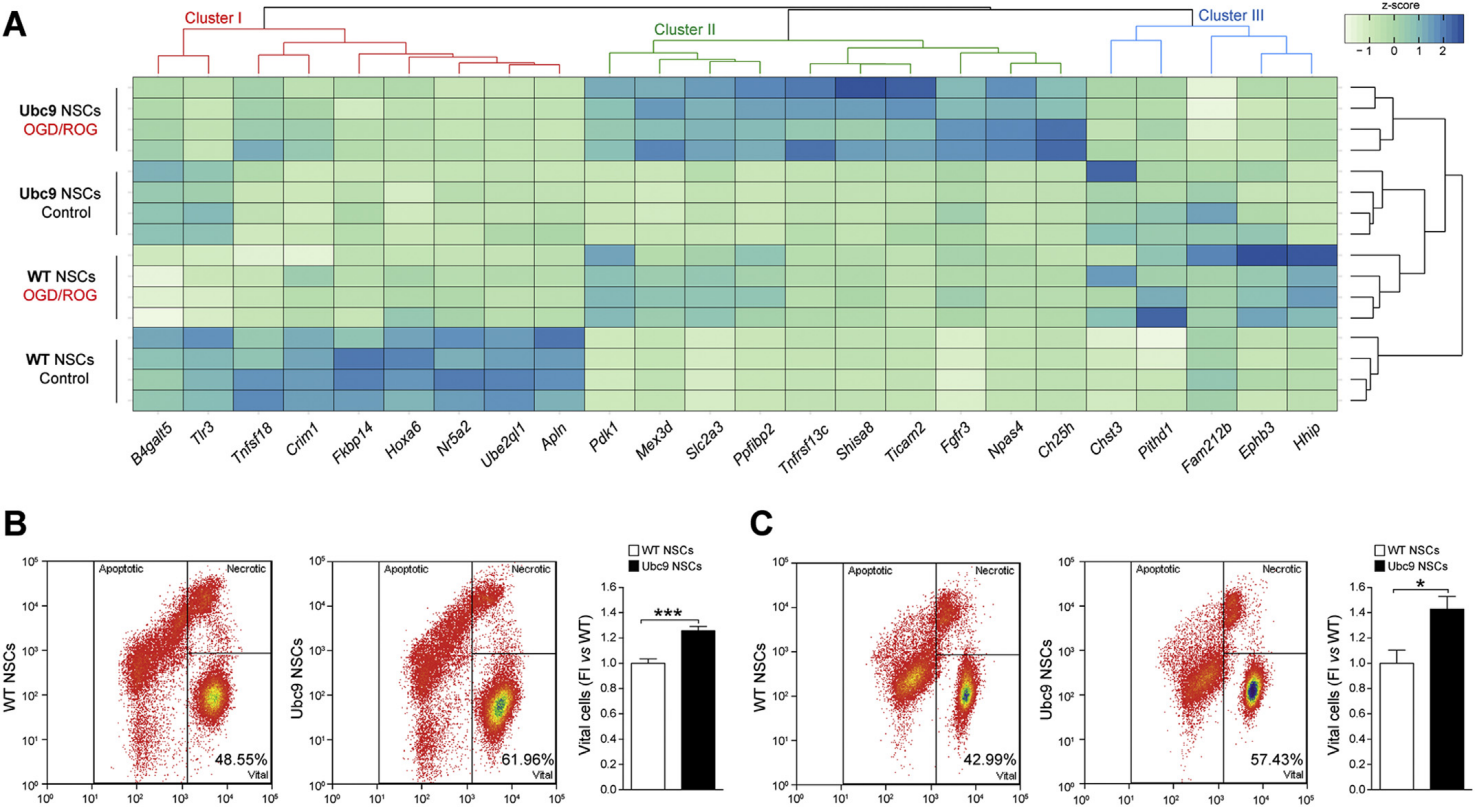
Ubc9 gain of function NSCs are protected against OGD/ROG *in vitro.* (A) Heatmap showing expression profile (z-score normalized intensities) of genes with a statistically-significant interaction (adjusted p-value<0.05) between the effect of OGD/ROG *vs* normoxia (control) and the effect of Ubc9 overexpression *vs* WT NSCs. The colored dendrogram shows the three expression clusters described in the text. See also Fig. S2, S3, S4 and Data S3. (B) Representative density plot and quantification of WT and Ubc9 undifferentiated NSCs post-OGD/ROG for live/dead analysis. Data are means ± SEM. *N* = 11 per group from 3 independent experiments, ***p-value<0.001 (2-tailed Student's t-test). (C) Representative density plot and quantification of WT and Ubc9 differentiated NSCs post-OGD/ROG for live/dead analysis. X axis (Hoechst blue), Y axis (PI). Data are means ± SEM. *N* ≥ 8 per group from 3 independent experiments, *p-value<0.05 (2-tailed Student's t-test). (For interpretation of the references to color in this figure legend, the reader is referred to the web version of this article.)

OGD/ROG induces drastic changes of genes belonging to several metabolic and biosynthetic pathways, including the hypoxia-inducible factor (HIF)-1α and the mitogen-activated protein kinase (MAPK) pathways (Fig. S3 and Data S2). Hence, to identify only those genes that responded differently to OGD/ROG in Ubc9 NSCs only, we applied an interaction model that tested the null hypothesis of no difference in fold changes after OGD/ROG (Fig. 3A).

Using this approach, we identified several genes with significantly different behaviors after OGD/ROG (Fig. 3A and Data S3), which altogether led to the identification of three main clusters of gene regulation (Fig. S4).

We defined cluster I as genes significantly downregulated in post-OGD/ROG WT NSCs (Fig. 3A, red, and Fig. S4). Cluster II includes genes that show a significantly stronger upregulation in post-OGD/ROG Ubc9 NSCs (Fig. 3A, green, and Fig. S4). Cluster III comprises genes upregulated in WT NSCs but downregulated in Ubc9 NSCs after OGD/ROG (Fig. 3A, blue, and Fig. S4).

In cluster I, we identified the *Cysteine Rich Transmembrane BMP Regulator 1* (*Crim1*), implicated in motor neuron development and survival [18]; the *TNF Superfamily Member 18* (Tnfsf18*)* [19]; and the *Homeobox A6* (Hoxa6), which regulates motor neuron identity [20] and inhibits apoptosis through the Bcl-2 signaling pathway [21]. Although the expression of these genes appears to be already different in WT and Ubc9 NSCs in normoxia (control), the increased expression after OGD/ROG in Ubc9 NSCs might be predictive of an enhanced neurogenic potential in conditions of metabolic distress.

In cluster II, we found genes linked with protective/adaptive responses after hypoxia [*i.e.* the *Toll Like Receptor Adaptor Molecule 2* (*Ticam2*) [22], *Shisa8* and *Slc2a3* (or GLUT3)], NSCs/neuronal survival [*i.e.* the *Fibroblast growth factor receptor 3* (*Fgfr3*), the *Cholesterol 25-Hydroxylase* (*Ch25h*), the *Neuronal PAS Domain Protein 4* (*Npas4*), and the *TNF Receptor Superfamily Member 13C* (*Tnfrsf13c* or *BAFF Receptor*)], neural plasticity [*i.e.* the *PPFIA Binding Protein 2* (*Ppfibp2*)] and cell homeostasis [*i.e.* the *Mex-3 RNA Binding Family Member D* (*Mex3d*), *Ch25h,* and *Npas4*].

Cluster III included the *Ephrin Receptor B3* (*EPhb3)*, the *PITH Domain Containing 1* (*Pithd1*), and the *Family With Sequence Similarity 212 Member B* (*Fam212*). *Pithd1* and *Fam212b* are regulators of cell function*,* the former being an activator of internal ribosomal entry site [23] and the latter being involved in cell energy homeostasis [24]. Thus, the decreased expression of these genes in Ubc9 NSCs might underlie a reduction of cell machinery and metabolic demands after OGD/ROG.

Structural replacement of damaged neural cells is one of the aims of exogenous NSC therapies in stroke. As such, we investigated gene expression changes in differentiated Ubc9 *vs* WT NSCs after OGD/ROG. We found that only two genes were upregulated in Ubc9 Diff after OGD/ROG (Data S3): *TICAM2* and the *Ankyrin Repeat Domain 1* (*ANKRD1*) (Fig. S5). *TICAM2* is also increased after OGD/ROG in undifferentiated Ubc9 NSCs (Fig. S5). Instead, the increase of *ANKRD1* is novel and in line with the evidence of its putative role in favoring anti-apoptotic and adaptive responses after ischemia [25].

Thus, OGD/ROG induces gene expression profile changes that are different between Ubc9 and WT NSCs, with increased transcripts related to protective/adaptive responses and reduced transcriptional activators after hypoxia. In fact, when assessed for resistance to OGD/ROG *in vitro*, both undifferentiated and differentiated Ubc9 NSCs showed significant increased survival compared to WT NSCs (Fig. 3B-C).

Our findings link the observed changes in gene expression induced by the Ubc9 transgene with an increased tolerance to conditions of scarce substrate (*i.e.* oxygen and glucose) availability *in vitro*.

### 3.4 Transplanted Ubc9 NSCs display increased survival and form more neurons in the stroke brain

We finally sought to validate our *in vitro* findings of tolerance to OGD/ROG by employing an *in vivo* model of ischemia/reperfusion injury.

Ubc9 and WT NSCs were transduced *in vitro* with a third-generation lentiviral vector coding for the farnesylated green fluorescent protein (GFP) and 1 × 10^5^ cells were transplanted intraparenchymally into the ischemic lesion, 72 h post-MCAO in mice.

**Fig. 4:**
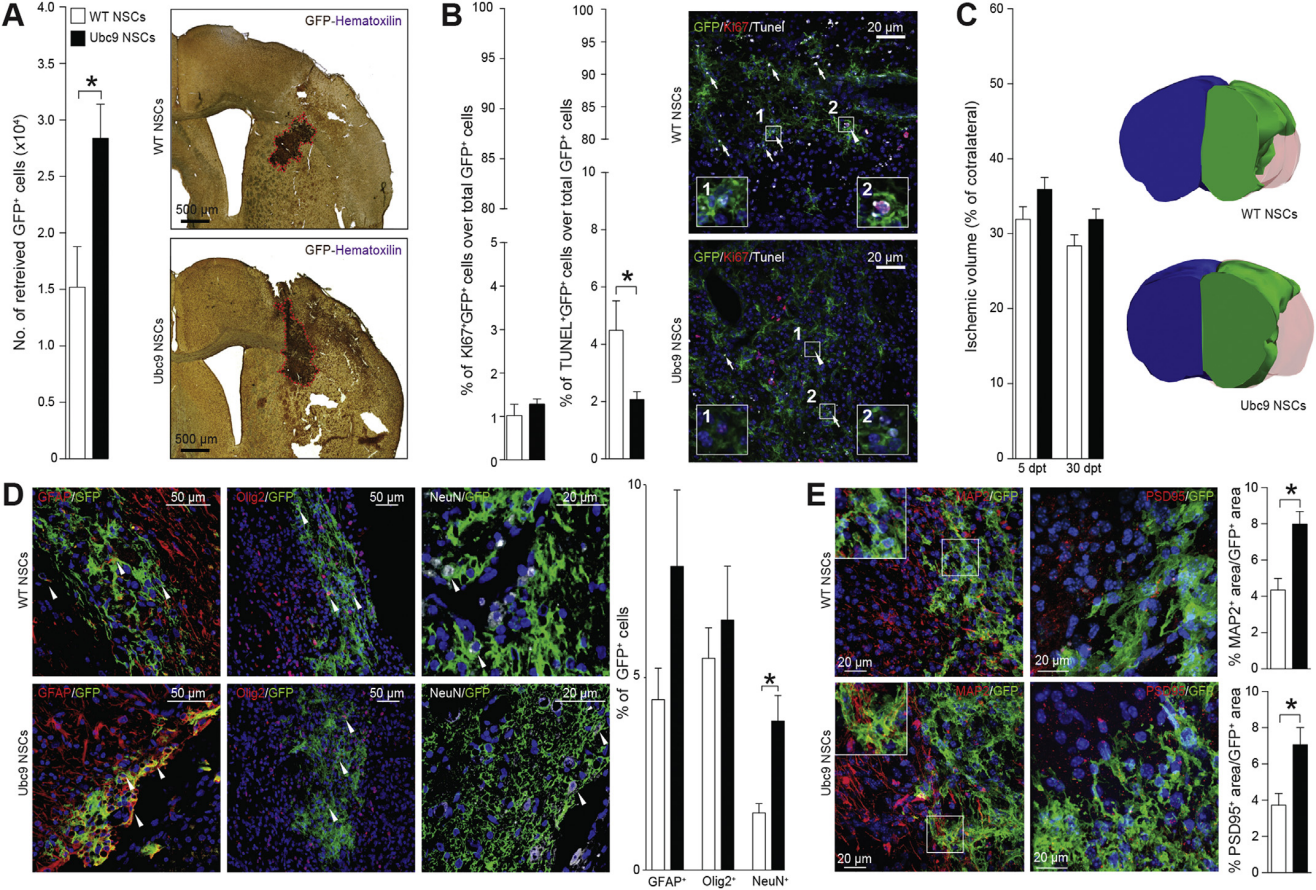
Transplanted Ubc9 NSCs display increased survival and form more neurons in the stroke brain. (A) Stereological quantification and representative IHC images of transplanted GFP^+^ WT and Ubc9 NSCs at 5 dpt revealing greater survival of Ubc9 NSCs. *N* ≥ 3 per group. (B) Quantification and representative images of Ki67^+^ and TUNEL^+^ transplanted GFP^+^ cells at 5 dpt. N ≥ 3 per group. Nuclei are stained with DAPI. (C) Representative 3D reconstructions of ischemic brains at 30 days post-transplantation (dpt) of mice transplanted with WT or Ubc9 NSCs (blue: contralateral hemisphere, green: healthy ischemic ipsilateral hemisphere, red: ischemic lesion). Data at 5 and 30 dpt are expressed as percentage of contralateral hemisphere. N ≥ 3 per group. (D) Representative images and quantification of GFP^+^ WT and Ubc9 NSCs stained for GFAP, OLIG2, and NeuN retrieved at 30 dpt within the ischemic hemisphere. Significantly more numerous GFP^+^ Ubc9 NSCs were found to be NeuN^+^ compared to GFP^+^ WT NSCs. (E) Representative images and quantification of GFP^+^ WT and Ubc9 NSCs stained for MAP2, and PSD95 retrieved at 30 dpt within the ischemic hemisphere. Significantly higher MAP2 and PSD95 expression is found in GFP^+^ areas, suggesting an increase of dendrite formation and integration of synapses, respectively. Nuclei are stained with DAPI. Data in A-E are means ± SEM. *p-value<0.05 (2-tailed Student's t-test). (For interpretation of the references to color in this figure legend, the reader is referred to the web version of this article.)

Since most transplanted stem cells die within the first few days after transplantation into the peri-ischemic environment [26], we assessed the survival of the NSC grafts sub-acutely at 5 days post-transplantation (dpt). Stereological analysis of the NSC grafts showed that 28.4 ± 3.0% of Ubc9 NSCs survived, compared to 15.2 ± 3.6% of WT NSCs (1.9-fold change) (Fig. 4A). This effect was not dependent on graft proliferation, but rather was related to the significantly-decreased cellular death of Ubc9 NSC grafts, as suggested by quantification of Ki67^+^ and TUNEL^−^ surviving Ubc9 NSCs (Fig. 4B).

We then characterized the long-term differentiation capacity of transplanted Ubc9 NSCs *in vivo*. Although no significant differences were found in total lesion volume at 5 or 30 dpt between ischemic mice transplanted with WT or Ubc9 NSCs (Fig. 4C), we found that the number of transplanted Ubc9 NSCs differentiating into neurons at 30 dpt was significantly higher (Fig. 4D). We also confirmed an increased expression in microtubule-associated protein (MAP) 2 and postsynaptic density protein (PSD) 95 in Ubc9 NSC grafts at 30 days post-transplantation, thereby suggesting an increase of dendrite formation and integration of synapses, respectively [27] (Fig. 4E). Of note, no signs of tumor formation caused by the grafted NSCs were ever detected in transplanted mice.

These data suggest that upregulating SUMOylation *via* provision of the Ubc9 transgene may be a novel approach to cellular preconditioning that has the potential to increase engraftment, survival, and neuronal differentiation of transplanted NSCs.

## 4 Discussion

Transplantation of NSCs has been extensively investigated in animal models of ischemic stroke [28]. However, methods for NSC delivery to the ischemic lesion are inherently flawed: while most NSCs distribute to peripheral organs when given systemically, the vast majority of transplanted cells die when directly injected into the brain [29]. Only 2% to 30% of transplanted NSCs survive after intraparenchymal transplantation in murine models of ischemic stroke, depending on the time of delivery [6]. While subacute delivery is the most effective in reducing clinicopathological deficits from stroke, it unfortunately also leads to the lowest survival of cellular grafts [6,29].

Several methodologies have been refined to enhance the survival of NSCs grafts within damaged CNS tissues [30]. These techniques include pre-conditioning cells prior transplantation (*i.e.*, exposing NSCs to reduced oxygen-tension, electrical fields, small molecules, or cytokines), the encapsulation of the cellular grafts within polymers or scaffolds, and the selective stable genetic engineering of NSCs [7]. While this latter approach has been used in the past to upregulate specific proteins/pathways [31,32], we took advantage of a multifaceted biological process, global SUMOylation, which preserves cellular functions and homeostasis under ischemic stress [33].

Using an unbiased protein and gene expression profiling of undifferentiated NSCs and their differentiated progeny, we show for the first time that SUMOylation regulates key aspects of NSC biology.

Increasing SUMOylation in undifferentiated NSCs led to a significant reduction in the expression of genes related to apoptosis and, upon differentiation, efficiently reduced cellular metabolic demands while upregulating genes involved in protective/adaptive responses. This is in line with previous evidence showing that SUMOylation has multiple substrates that control mitochondrial metabolism and mitochondria-dependent apoptosis [34]. Among these, SUMOylation of dynamin-related protein 1 (DRP1) leads to a stabilisation of its active pool with increased mitochondrial fission [35], which is linked to reduced respiratory activity and lower energy demands [36]. After OGD/ROG, drastic changes of several metabolic and biosynthetic pathways, including HIF-1α and MAPK, appear in NSCs. SUMOylation increases the expression of genes linked with adaptation to hypoxia (cluster I and II), while reducing genes that act as activators of cellular functions (cluster III). Altogether, these genetic and metabolic changes lead to increased resistance to OGD/ROG in both undifferentiated and differentiated Ubc9 NSCs *in vitro*, as well as a ~2-fold increase in the survival of NSC grafts *in vivo.*

We found that increased SUMOylation in NSCs confers a higher predisposition to differentiate into neurons both *in vitro* and *in vivo*. This is a unique advantage of our approach, which could be key for the treatment of CNS disorders characterized by neurotoxicity and/or neurodegeneration. The finding that most of the upregulated genes in undifferentiated Ubc9 NSCs have a role in neurogenesis and neuronal function is well in line with the prevalent expression of Ubc9 in neuron-dense brain areas [34,37]. SUMOylation is indeed one of the master regulators of neuronal function through regulating the activity of several transcription factors, including the topoisomerases I and II, p53, and members of the myocyte enhancer factor 2 (MEF2) family [34,37]. Critically, the SUMOylation of Sox2 negatively regulates its transcriptional roles *via* impaired binding to DNA [17]. In fact, we documented an increased SUMOylation of Sox2 in Ubc9 NSCs by IP. Interestingly, we also found that key genes involved in neuroprotection were upregulated in undifferentiated and differentiated Ubc9 NSCs after OGD/ROG, which might confer a more-resilient phenotype to NSC-derived neurons in the stroke brain.

Finally, we show that SUMOylation plays a role in cell proliferation and cycle in NSCs, leading to higher growth rates and self-renewal *in vitro.* The consequences of these effects were negligible *in vivo*, as we found that the fraction of proliferating cells was similar between mice grafted with Ubc9 NSCs and WT NSCs*,* with no evidence of mass formation/neoplasia in either group. This is in line with novel evidence suggesting that SUMO acts on chromatin to stabilize key determinants of somatic and pluripotent states [38], and suggests that increasing SUMOylation will safely boost the multifactorial regenerative potential of NSCs.

Overall, our data suggest that upregulating SUMOylation is a valuable strategy for improving the efficacy of experimental NSC-based therapies for ischemic stroke *via* induction of a homeostatic balance between the graft and the ischemic microenvironment.

Future work will seek to employ alternatives to genetic manipulations of the graft, such as preconditioning with small molecules [[39], [40], [41]], to upregulate global SUMOylation prior to transplants.

## Author contributions

Conceptualization and methodology: J.D.B. and L.P.J.; Generation and analysis of data: J.D.B., L.P.J., T.L., N.V., D.Y., K.R.J., D.M., Y.M., A.B., M.W. and Y.L.; Writing – Original Draft: J.D.B. and L.P.J.; Writing – Review and Editing: G.K.F., R.J.M., J.M.H. and S.P.; Funding Acquisition: J.M.H. and S.P.; Resources: J.M.H. and S.P.; Supervision: R.J.M., J.M.H. and S.P.

## Conflict(s) of interest/disclosures

J.D.B. has equity in CITC Ltd. and Avidea Technologies; S.P. is co-founder and CSO at CITC Ltd.

## Appendix A Supplementary data

Supplementary material 1 Image 1

Supplementary material 2 Image 2

Supplementary material 3 Image 3

Supplementary material 4 Image 4
