# Supplementary material for: SUMOylation promotes survival and integration of neural stem cell grafts in ischemic stroke
Source: eBioMedicine. 2019 Mar 21;42:214–24. doi: 10.1016/j.ebiom.2019.03.035 (PMC6491415; doi:10.1016/j.ebiom.2019.03.035)
Supplement: Supplementary file 4 — Supplementary material 4 [file mmc4.pdf]

SUPPLEMENTAL FIGURES AND DATA

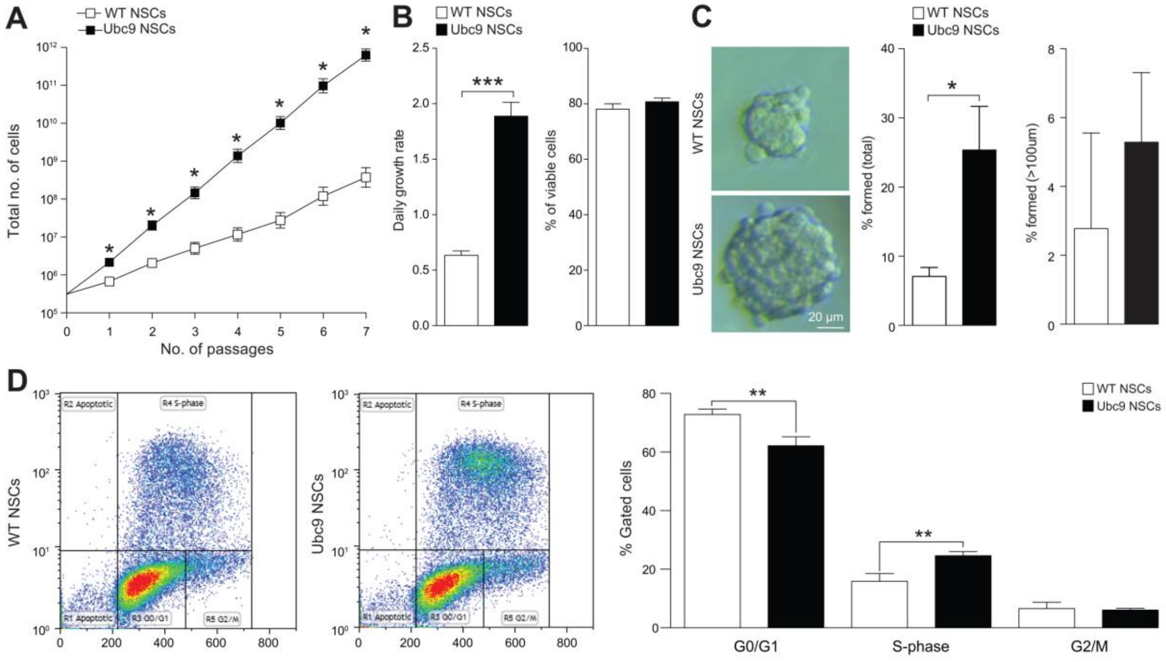

Figure S1. Ubc9 NSCs show higher growth rate, clonogenicity and increased cell cycle S phase *in vitro*.

Related to Figure 1.

(A) Linear growth curves of Ubc9 NSCs and WT NSCs over 7 passages *in vitro*. Data are means ± SEM. N≥ 3 independent experiments.

(B) Ubc9 NSCs display a significantly faster growth rate, but similar viability, compared to WT NSCs *in vitro*. Data are means ± SEM. N≥ 3 independent experiments.

(C) Ubc9 NSCs demonstrate significantly greater clonogenicity (expressed as % of formed spheres) *vs* WT NSCs. No significant differences in sphere size (>100 um) are seen. Data are mean ± SEM. N≥ 3 independent experiments.

(D) Representative FACS plots for cell cycle analysis of WT and Ubc9 NSCs and quantifications. Ubc9 NSCs show a significant increase in cells in S-phase and decrease in cells in G0/G1-phase compared to WT NSCs. N≥ 3 independent experiments.

Data in A-D are means ± SEM shown. *p-value< 0.05 and ***p-value< 0.001 (2-tailed Student's t test).

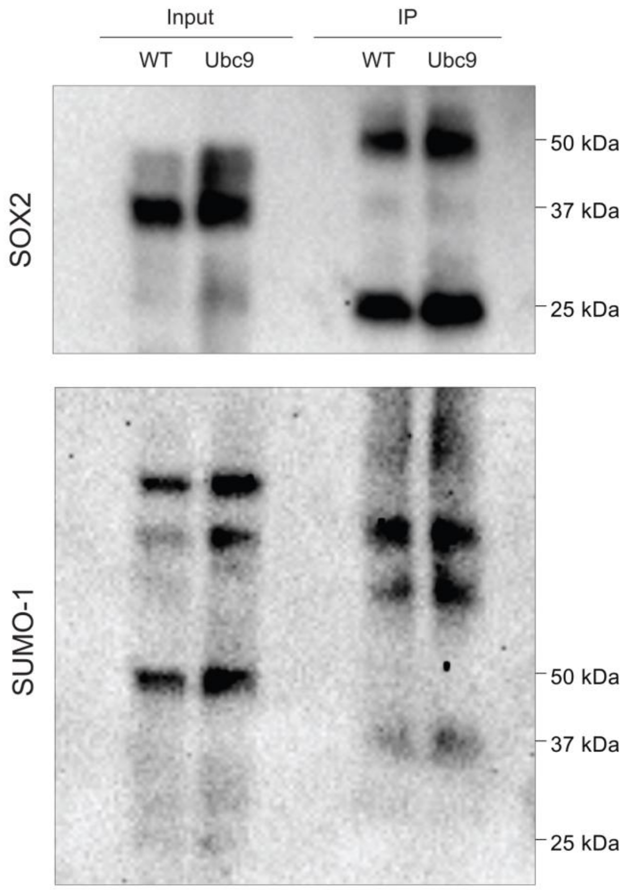

Figure S2. Ubc9 NSCs show higher SUMOylation of the transcription factor Sox2.
Representative immunoblot of immunoprecipitation (IP) for Sox2 in WT NSCs and Ubc9 NSCs probed with an anti-Sox2 antibody (upper panel) and an anti-SUMO-1 antibody (lower panel). Ubc9 NSCs show increased SUMO-1 reactivity in the input and in the IP lanes, including at the level of Sox2 (expected MW of 37kDa).
Equal amounts of proteins were loaded, bands at 25kDa and 50kDa in the upper panel represent the light and heavy antibody chains used in the IP.
Input= precleared lysate, IP= immunoprecipitated lysate.

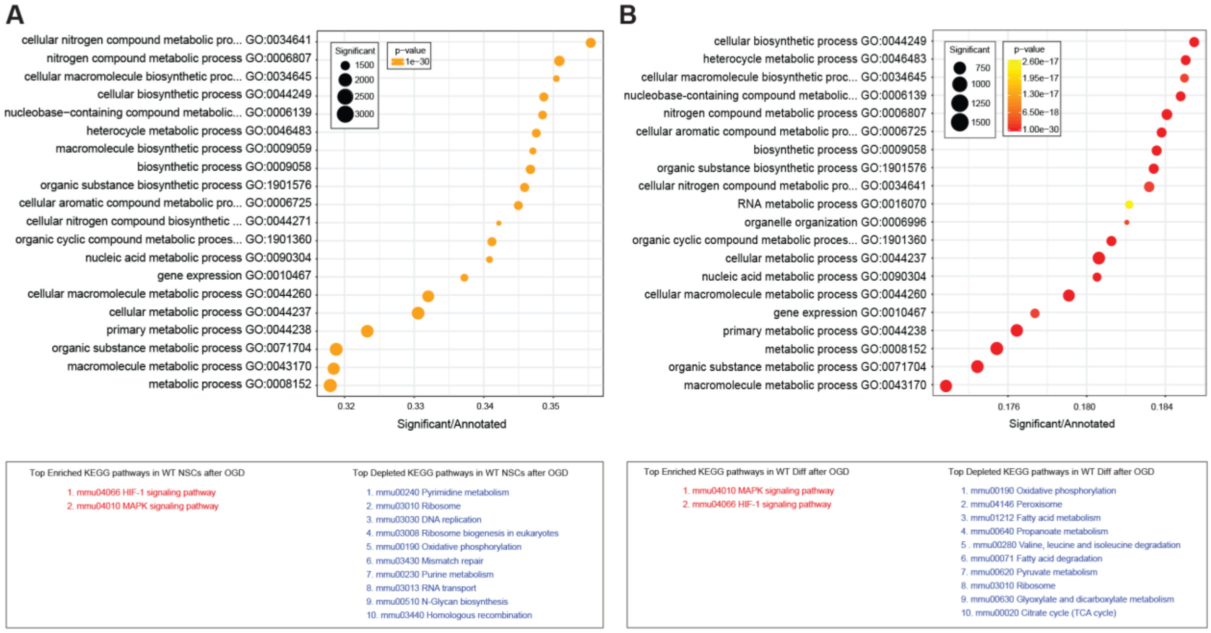

Figure S3. Effect of OGD/ROG on the gene expression profile of WT NSCs *in vitro*. Related to Figure 3 and Data S2.

Plots of the GO enrichment results for genes differentially expressed in undifferentiated (A) and differentiated (B) WT NSCs after OGD/ROG *vs* control WT NSCs. The x-axis shows the enrichment ratio, the colour indicates the enrichment p-value (Kolmogorov–Smirnov statistic) and the size of each point indicates the number of significant genes in the corresponding GO category. The tables below the plots show the KEGG pathways showing the strongest enrichment (GAGE analysis p-value< 0.05 for all pathways) for upregulated (red) and downregulated (blue) genes in the same comparison.

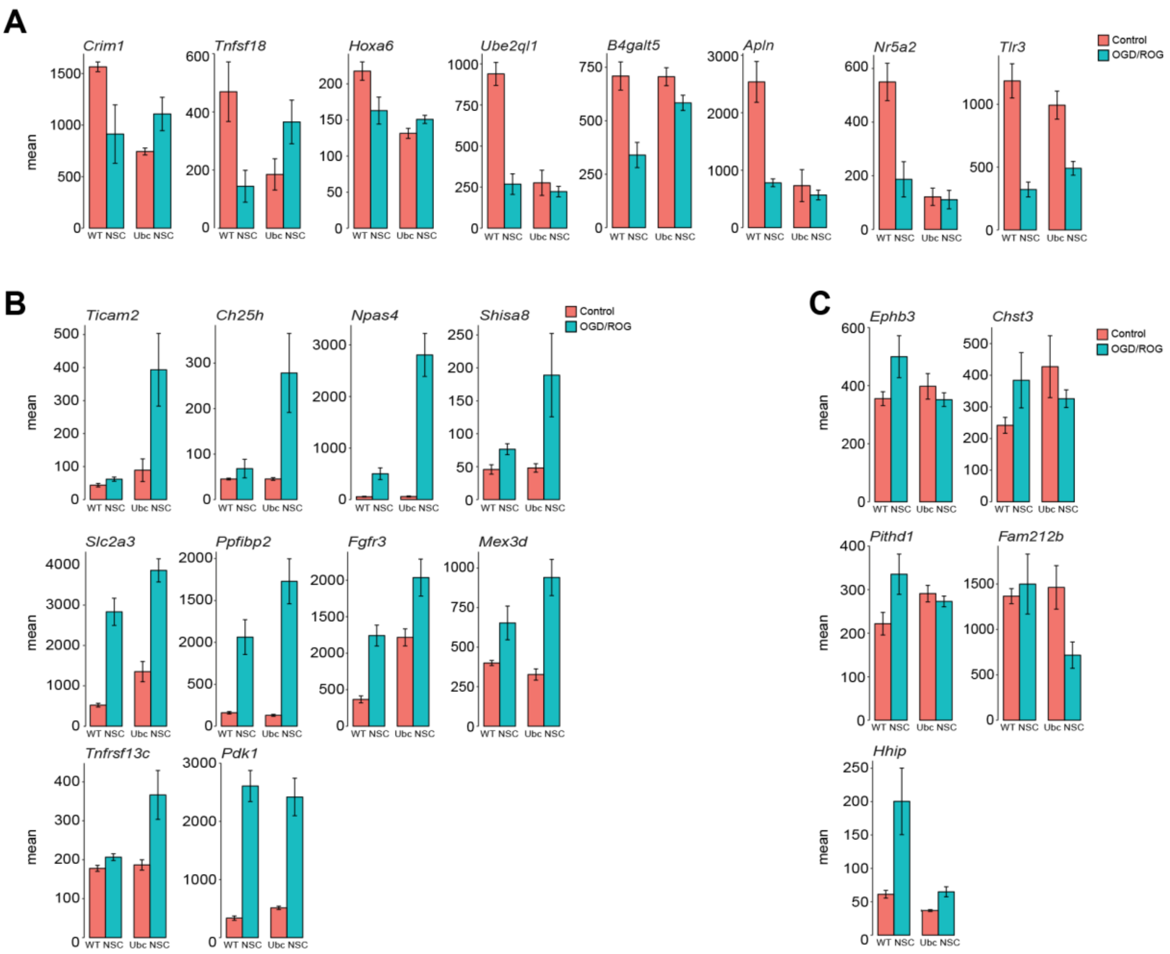

Figure S4. Microarray expression analysis of cellular transcripts upon OGD/ROG in undifferentiated WT NSCs and Ubc9 NSCs.

Related to Figure 3 and Data S3.

Expression profile of genes with a significant interaction coefficient (adjusted p-value < 0.05) between response to OGD/ROG and genotype (Ubc9 NSCs *vs* WT NSCs). The bars show the mean microarray intensity ± SEM. (A) Cluster I, genes significantly more downregulated upon OGD/ROG in WT NSCs than in Ubc9 NSCs. (B) Cluster II, genes showing a significantly stronger upregulation upon OGD/ROG in Ubc9 NSCs than in WT NSCs. (C) Cluster III, genes undergoing downregulation in Ubc9 NSCs after OGD/ROG, but upregulation in WT NSCs after OGD/ROG.

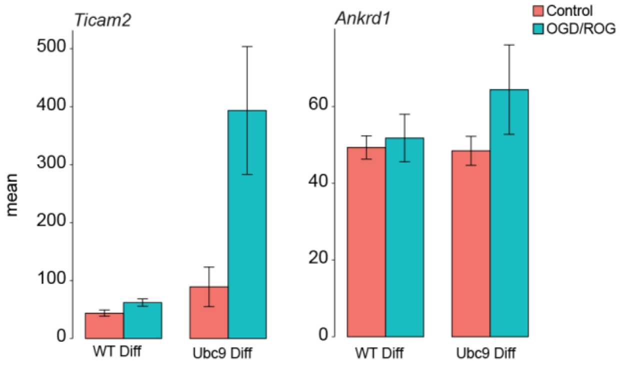

Figure S5. Microarray expression analysis of cellular transcripts upon OGD/ROG in differentiated WT and Ubc9 NSCs.
Related to Figure 3 and Data S3.
Expression profile of genes with a significant interaction coefficient (adjusted p-value <0.05) between response to OGD/ROG and genotype (Ubc9 Diff *vs* WT Diff). The bars show the mean microarray intensity ± SEM.

Data S1. Protein target identification via liquid chromatography (LC)/mass spectrometry (MS) analysis of Ubc9 NSCs and WT NSCs after global SUMO-1 immunoprecipitations (IPs).
Related to Figure 1D-E.

Data S2. Differential expression analysis for microarray experiment.
Data from the following comparisons are shown:
Ubc9_NSCs_*vs*_WT_NSCs,
Ubc9_Diff_*vs*_WT_Diff,
WT_NSC_OGD_*vs*_CTRL,
WT_Diff_OGD_*vs*_CTRL,
Ubc9_NSCs_OGD_*vs*_CTRL,
Ubc9_Diff_OGD_*vs*_CTRL,
Ubc9_NSC_OGD_*vs*_WT_NSCs_OGD,
Ubc9_Diff_OGD_*vs*_WT_Diff_OGD/
(CTRL: control, i.e. same cell line without OGD/ROG).
Related to Figure 1F-H, 2A-C, S2.

Data S3. Interaction analysis between treatment and genotype for microarray data.
Related to Figure S3, S4.
